# *Capnocytophaga* Induced Acute Necrotizing and Exudative Pericarditis with Abscess Formation

**DOI:** 10.1155/2018/6437928

**Published:** 2018-08-15

**Authors:** Alexa Bello, Alejandro Castaneda, Abhay Vakil, Joseph Varon, Salim Surani

**Affiliations:** ^1^Universidad Popular Autonoma del Estado de Puebla, Puebla, PUE, Mexico; ^2^Dorrington Medical Associates, Houston, TX, USA; ^3^Universidad Autonoma de Baja California, Houston, TX, USA; ^4^University of North Texas, Denton, TX, USA; ^5^The University of Texas Health Science Center at Houston, Houston, TX, USA; ^6^The University of Texas Medical Branch at Galveston, Galveston, TX, USA; ^7^United Memorial Medical Center, Houston, TX, USA; ^8^Division of Pulmonary, Critical Care & Sleep Medicine, Health Science Center, Texas A&M University, Corpus Christi, TX, USA

## Abstract

We present the case of a 55-year-old gentleman, with bilateral pulmonary embolism and a large pericardial effusion that lead to a pericardial window with evacuation of creamy pus. Gram stains were negative, with culture growing *Capnocytophaga*. Pathology revealed acute necrotizing and exudative changes, including frank abscess formation. In developed countries, pericardial abscess and acute pericarditis are uncommon due to availability of broad-spectrum antibiotics. Pericardial abscess due to *Capnocytophaga* is even more uncommon.

## 1. Introduction

Acute pericarditis (AP) represents nearly 0.1% of the diagnoses of patients being hospitalized for acute chest pain not related to myocardial infarction and nearly 5% of the diagnoses in the emergency department [1]. Although AP is common, its treatment represents a challenge due to delays in diagnosis, which increases the complications [2, 3]. Recurrences have been reported in nearly 30% of the patients who have experienced AP [2]. Acute pericarditis can be caused by infectious and noninfectious etiologies [1, 4]. In developed countries, purulent bacterial pericarditis is less frequent due to the availability of broad-spectrum antibiotics [4]. We present a case of purulent pericarditis with necrotizing and exudative changes leading to cardiac tamponade.

## 2. Case Presentation

A 55-year-old gentleman with a history of diabetes mellitus, hypertension, cocaine, and marijuana use presented to the emergency department (ED) with complains of chest pain and dyspnea for past 6–8 months, as well as lower extremity edema and weight loss. On admission to the hospital, his vital signs were stable. Physical examination revealed obesity, decreased breath sounds bilaterally, and mild tachycardia, and point of maximal impulse was enlarged and displaced at the presence of edema on bilateral lower extremities. The patient has poor dentition with cavity in the left second molar tooth. The rest of the examination was otherwise unremarkable. Complete blood count revealed a hematocrit of 33.1%, hemoglobin 9.7 g/dL, platelet count 232,000/mL, and white blood count 8.6 × 10^3^. Blood chemistry was unremarkable. B-type natriuretic peptide was 613 pg/mL.

**Figure 1:**
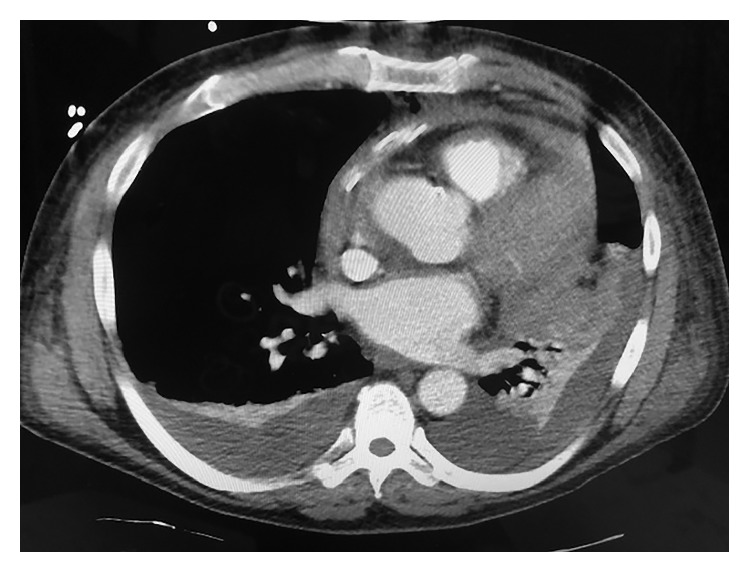
Computed tomography (CT) scan of the chest with intravenous contrast, which reveals a very large pericardial effusion, compressing the right and left ventricles and the right atrium.

**Figure 2:**
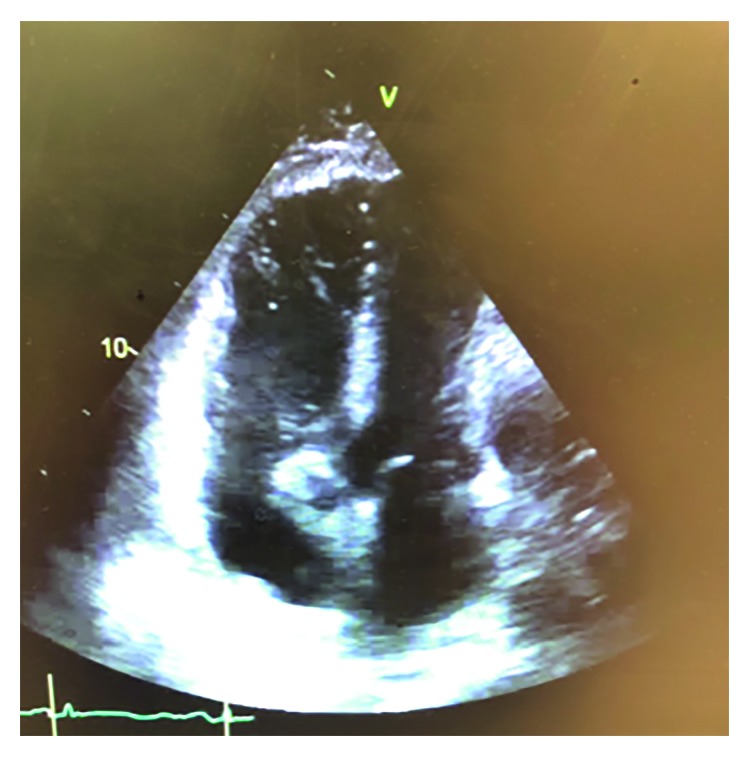
2D echocardiogram image of cardiac tamponade with right ventricular diastolic collapse and a large fibrinous exudative pericardial effusion.

**Figure 3:**
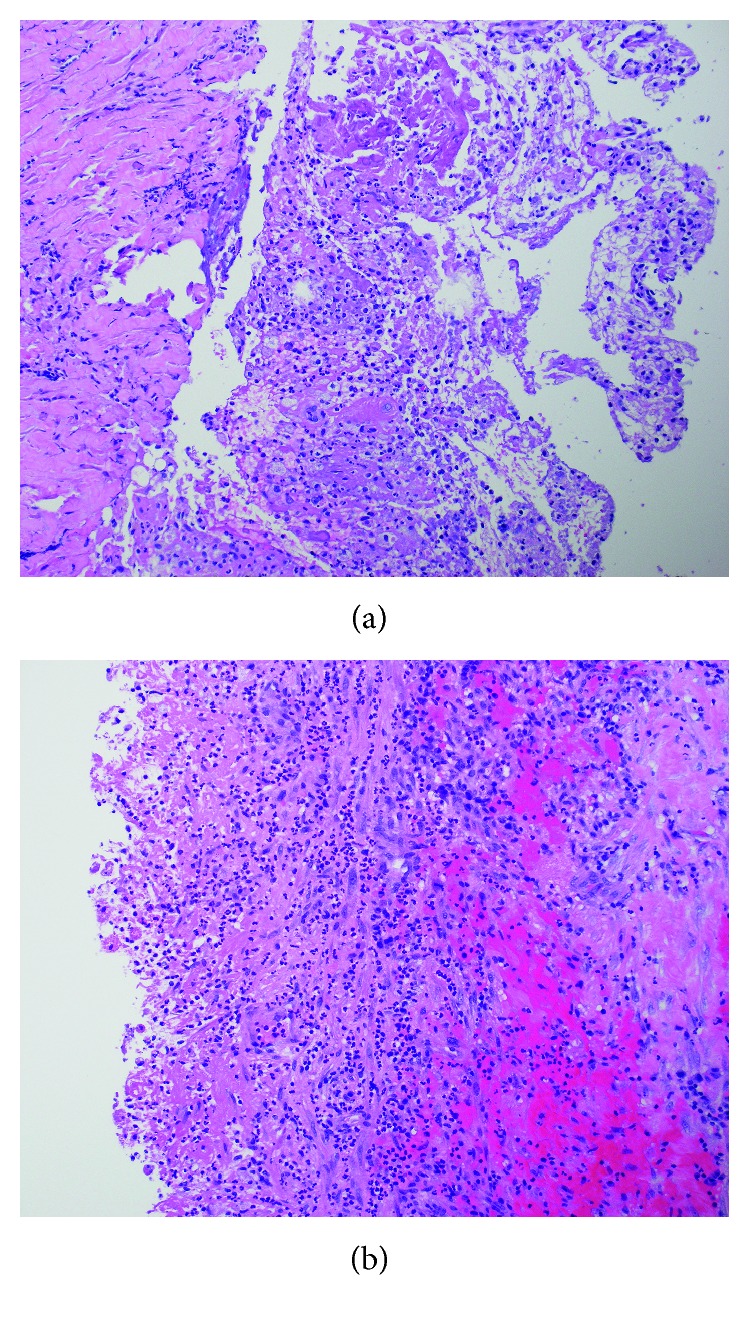
Low power and high power of pericardial tissue showing acute necrotizing and exudative changes and frank abscess formation. (a) Low-power view. (b) High-power view.

Because of his chest pain and associated signs, the patient underwent a computed tomography (CT) scan of the chest with intravenous contrast, which revealed a very large pericardial effusion, compressing the right and left ventricles and the right atrium (Figure 1). In addition, there was consolidation in the left mid lung, bilateral pleural effusions, and bilateral pulmonary embolism. A 2D echocardiogram revealed cardiac tamponade with right ventricular diastolic collapse, with a large fibrinous exudative pericardial effusion (Figure 2). The patient underwent an emergent pericardial window due to his clinical signs and symptoms consistent with cardiac tamponade. The pericardial drainage showed a significant amount of yellow creamy pus with thickened pericardium. Anaerobic culture reported the presence of *Capnocytophaga* species. The pathology specimen showed acute necrotizing and exudative changes including frank abscess formation with no specific organism detected and no evidence of malignancy (Figures 3(a) and 3(b)).

The patient's condition improved postoperatively and was placed on piperacillin and tazobactam for four weeks. His pulmonary embolism and acute deep vein thrombosis were treated with systemic anticoagulation. The patient was discharged home on apixaban and has been seen on the follow-up visit with significant improvement in his symptoms.

## 3. Discussion

Acute pericarditis remains a common condition in patients seen in the ED with a chief complaint of chest pain unrelated to myocardial infarction [1]. This entity results from inflammation of the pericardium, a fibrous tissue that surrounds the heart and the roots of the great blood vessels [1, 4]. Early recognition of AP is important in order to improve outcome for patients [3]. Due to its clinical similarity with various acute coronary syndromes and pulmonary embolism, early recognition is often dismissed [4].

When clinically recognizable, the symptomatology of AP mainly includes central and pleuritic chest pain and sharp retrosternal pain with common characteristics such as irradiation to one or both trapezius ridges, neck, jaw, or arms imitating myocardial ischemia with various degrees of severity [1, 5]. In addition, other manifestations include the presence of pericardial friction rub, pericardial effusion, and widespread ST elevation or PR depression in the electrocardiogram [5]. If this condition worsens, the syndrome may result in cardiac tamponade with subsequent constrictive pericarditis [6].

In this case, the patient was found to have AP as well as imaging findings. Chest CT showed a large pericardial effusion that was compressing the ventricles and the right atrium.

Our patient grew *Capnocytophaga*. The genus *Capnocytophaga* was first described in 1979 by Leadbetter and coworkers [7]. It belongs to the family Flavobacteriaceae and the phylum Bacteroidetes [8]. It is found in the oral flora of humans and animals [7, 8]. This genus has 9 species, 7 of them (i.e., *C. gingivalis*, *C. granulosa*, *C. haemolytica*, *C. leadbetteri*, *C. ochracea*, *C. sputigena*, and *Capnocytophaga* genospecies AHN8471) are part of the commensal bacteria in the oral flora of humans, which are capable of causing sepsis in immunocompromised patients [9]. *C. canimorsus* and *C. cynodegmi* are found mostly in the bacterial oral flora of dogs and cats [8, 9].

In our patient, there was no history of recent animal bite. His sepsis was due to immunocompromise. Studies have shown that *Capnocytophaga* bacteremia is associated with severe oral pathology and neutropenia, which can also result in abscess, fulminant sepsis, lung abscess, endocarditis, and meningitis [9–12]. Mortality reaching up to 30% for septicemia and approximately 5% for meningitis has been reported [13].

The identification of *Capnocytophaga* species is difficult except by DNA hybridization [10]. This genus is not detected by the innate immune system; therefore, a proinflammatory response is not initiated, which results in the inability of toll-like receptor 4 to respond to *Capnocytophaga*. In addition, interleukin-6, interleukin-8, nitric oxide, tumor necrosis factor-a, and other proinflammatory cytokines are absent [8]. The inability of complement killing as well as to polymorphonuclear-mediated phagocytosis of this genus leads to a rapid multiplication that can cause general sepsis, as seen in our patient, or deadly shock if not treated correctly [13].

The first-line treatment for this clinical entity is penicillin, followed by amoxicillin-clavulanate or third generation cephalosporin. However, antibiotics such as imipenem, clindamycin, and doxycycline have also shown clinical benefits [9]. Our patient was maintained on piperacillin and tazobactam rather than switching to penicillin as he was responding to the therapy adequately.

## 4. Conclusion

An early diagnosis and approach for AP benefits the outcome of patients avoiding complications. In our case, acute necrotizing purulent pericarditis induced by *Capnocytophaga* species was successfully treated.
